# Evaluation of Physical Therapy Interventions for Improving Musculoskeletal Pain and Quality of Life in Older Adults

**DOI:** 10.3390/ijerph19127038

**Published:** 2022-06-08

**Authors:** Soraya Pacheco-da-Costa, Concepción Soto-Vidal, Victoria Calvo-Fuente, María José Yuste-Sánchez, Beatriz Sánchez-Sánchez, Ángel Asúnsolo-del-Barco

**Affiliations:** 1 Neuromusculoskeletal Physical Therapy in Stages of Life Research Group (FINEMEV), Department of Nursing and Physical Therapy, Faculty of Medicine and Health Sciences, Universidad de Alcalá, Autovia A2, km 33.200, 28805 Alcalá de Henares, Madrid, Spain; soraya.pacheco@uah.es (S.P.-d.-C.); victoria.calvo@uah.es (V.C.-F.); 2 Physiotherapy in Women’s Health Research Group (FPSM), Department of Nursing and Physical Therapy, Faculty of Medicine and Health Sciences, Universidad de Alcalá, Autovia A2, km 33.200, 28805 Alcalá de Henares, Madrid, Spain; marijo.yuste@uah.es (M.J.Y.-S.); beatriz.sanchez@uah.es (B.S.-S.); 3 Public Health and Epidemiology Research Group (ISPE), Department of Surgery, Social and Medical Sciences, Faculty of Medicine and Health Sciences, Universidad de Alcalá, Autovia A2, km 33.200, 28805 Alcalá de Henares, Madrid, Spain; angel.asunsolo@uah.es

**Keywords:** quality of life, physical therapy, elderly, therapeutic exercise, therapeutic education program

## Abstract

Background: The ageing process may lead to functional limitations, musculoskeletal pain, and worsened quality of life. The aim of this paper is to evaluate two physical therapy interventions for reducing musculoskeletal pain and improving quality of life in older adults. Methods: A cohort study was carried out with older people (60–75 years old). The Geriatric Physical Therapy group (*n* = 70) received massage therapy, therapeutic exercise, and therapeutic education program for 5 weeks; the Standardized Therapeutic Exercise group (*n* = 140) received a standardized therapeutic exercise and therapeutic education program for 3 weeks. Health-related quality of life (SF-36v2) and musculoskeletal pain intensity (VAS) were collected at baseline (A_0_), post-intervention (A_1_), and 12 weeks after baseline (A_2_). Results: There was pain intensity reduction in both groups (*p* < 0.05) and health-related quality of life improvement, except for Emotional Role (*p =* 0.34); Physical Function (*p* = 0.07), Bodily Pain (*p* = 0.02), and General Health (*p* = 0.09). At A_2_ there was a difference (*p* < 0.05) for neck pain in favor of the Geriatric Physical Therapy Group. Conclusions: Within the limitations of the study, it was possible to conclude that both physical therapy interventions showed a positive effect for reducing non-specific neck pain and low back pain in older adults, which may contribute to health-related quality of life improvement.

## 1. Introduction

The increasing age of the population is a serious worldwide problem that Spain and other European Union countries are facing at the moment, and it sets the challenge of raising the opportunities for improving quality of life (QoL) of elderly people [1,2,3,4]. QoL is related to people’s degree of satisfaction with their physical and emotional status, family life, and loving and social matters. Health-related quality of life (HRQoL) improvement is associated with the general welfare of individuals and includes areas such as physical health, psychological status, level of autonomy and independence, personal relationships, beliefs, social level, and perceived social support [3,4,5].

The ageing process is often associated with the deterioration of various physiological capacities that can lead to functional limitations and loss of personal autonomy [1,2,3,4,5,6,7]. Age is also a well-known risk factor for musculoskeletal pain in association with gender, inactivity, social environment, and prior work exposure [3,4,5,6,7]. Physical inactivity and sedentary behaviors are important environmental factors for explaining the high prevalence of functional limitations, musculoskeletal pain, and worse HRQoL perception in older adults [5,6,7,8,9,10,11].

One of the most common reasons older people access primary care settings is musculoskeletal pain, especially non-specific chronic neck and low back pain [12,13,14,15,16]. Pain may decrease physical function and limit overall performance of daily living activities and may lead to a negative impact on HRQoL perception and functioning in older adults [17,18,19]. Some authors posit that structured physical activity, as well as therapeutic exercise, have a positive effect on preventing musculoskeletal pain, chronic diseases, and functional limitation, leading to better QoL perception [20,21,22,23,24].

The most common physical therapy interventions described in the literature for reducing musculoskeletal pain, especially non-specific chronic neck pain and/or low back pain and/or QoL improvement, include stretching, massage therapy, therapeutic exercise, and therapeutic education programs [15,16,24,25,26,27,28,29,30,31,32,33,34,35,36,37]. Moreover, the most effective interventions are those that combine the above-mentioned techniques [15,27,30,32].

Therefore, the aim of this paper is to evaluate two different physical therapy interventions, one including individualized sessions of stretching, massage therapy, and therapeutic exercise plus a therapeutic education program; and the other including standardized therapeutic exercise plus a therapeutic education program for reducing non-specific chronic neck and low back pain intensity and improving QoL perception in older adults. Furthermore, we compared the two different physical therapy interventions, namely individualized and standardized, in this population.

## 2. Materials and Methods

A prospective, non-randomized, cohort study was carried out at the Physical Therapy Unit, University of Alcalá, Spain. Ethical approval for the studies was obtained from the Principe de Asturias Hospital research and ethics committee (PI: 02/10) in Alcalá de Henares, Spain, and conformed to the ethical guidelines of the Declaration of Helsinki.

### 2.1. Sample

Study participants were consecutively recruited through print and e-mail advertising within University of Alcala workers, and at elderly leisure centers at Alcalá de Henares and surroundings.

Inclusion criteria were individuals between 60 and 75 years old; autonomous for Activities of Daily Living (ADL), confirmed through 100 points of the Spanish version of the Barthel Index [38]; and diagnosed with non-specific chronic neck and low back pain by their primary care physicians. Individuals who had a surgery 6 months before starting the study or who were diagnosed with a neurologic or musculoskeletal disease were excluded.

Sample size was calculated based on the detection of a minimal clinical important difference (MCID) in a minimum of 3 points in the Spanish version of the Short Form-36 version 2 Health Survey (SF-36v2) for measuring HRQoL [39,40,41], and a minimum 2 cm reduction in pain intensity measured with the Visual Analogue Scale (VAS) [42,43]. Therefore, the subjects who met the inclusion criteria were consecutively included in the study: 70 participants in the Geriatric Physical Therapy group (GPTG); and 140 individuals in the Standardized Therapeutic Exercise group (STEG). Since the STEG intervention was shorter, in order to minimize risk of bias, estimating a standardized mean difference of 0.4, with a significance level of 5% and a power of 80%, there were 2 people assigned to the STEG for each subject assigned to the GPTG.

Beforehand, individuals were informed about the study, and written informed consent was obtained from all participants.

### 2.2. Outcomes Measures

Sociodemographic and anthropometric variables, such as, age, sex, marital and work status, height, weight, and body mass index (BMI) were collected at baseline assessment (A_0_) for descriptive characteristics.

Primary outcomes, non-specific neck and low back pain, and HRQoL were collected in both groups at 3 different moments: baseline (A_0_), post-intervention (A_1_), and 12 weeks after baseline (A_2_).

HRQoL was measured with the Spanish version of SF-36v2, which has been culturally adapted and validated in the Spanish population [39] and has its population-based norms for people aged 60 to 85 and over [40]. This questionnaire has 36 items divided into 8 dimensions of health status, such as Physical Function (PF), Physical Role (PR), Body Pain (BP), General Health (GH), Vitality (VT), Social Function (SF), Emotional Role (ER), and Mental Health (MH). Each of the dimensions is scored from 0 (worst health status perception) to 100 (best health status perception). Moreover, there are values for Physical Component Summary (PCS) and Mental Component Summary (MCS) that are a combination of the results in different items and dimensions. The MCID described for SF-36v2 is a minimum of 3 points in the dimensions [41].

Non-specific neck and low back pain intensity were measured with VAS. This simple instrument quantifies pain intensity numerically in centimeters from 0 (no pain) to 10 (worst pain), and it requires a degree of understanding and cooperation from the patient. It shows good correlation with the descriptive scales, good responsiveness and reliability, and it is easily reproducible. A difference of 2 centimeters in VAS is the value considered as MCID [42,43].

### 2.3. Interventions

Both interventions were part of the “Physical Therapy workshop for older adults” at the University of Alcala, Spain. Both group interventions were designed and delivered by 5 Physical Therapy Degree professors, who are physical therapists with over 10 years of experience in treating patients with neck or low back pain. Participants in both groups received each group intervention with no more than 10 participants in each session.

All the materials used for both group interventions, such as brochures, booklets, pain intensity diaries, and slide projections, were elaborated by the researchers and are available in Spanish from the authors.

#### 2.3.1. Geriatric Physical Therapy Group

Table 1 shows the physical therapy intervention for the GPTG that was carried out for 5 weeks and a follow-up at week 12. Participants with non-specific neck pain received individualized sessions of massage therapy as described by Sefton et al. [29] and therapeutic exercise as described by Häkkinen et al. [26]; participants with non-specific low back pain received individualized sessions of massage therapy as described by Cherkin et al. [34] and therapeutic exercise as described by Albaladejo et al. [15]. At the end of each session, there was a group activity for the Therapeutic Education Program on educational and preventive measures to improve health status that included different activities and conferences about the relation between being physically active, therapeutic exercise routines, QoL improvement, postural hygiene, risk of falls, and pain neuroscience education [24,25].

Each participant received a brochure with a detailed description of the individualized Therapeutic Exercise Program developed during the sessions, which allowed individuals to perform the home based individualized Therapeutic Exercise Program for 30 min, 4 times a week. The compliance of the home-based exercise program was measured through a diary that the participants had to fulfil each time they performed it at home, reporting how they felt before and after doing it.

#### 2.3.2. Standardized Therapeutic Exercise Group

Table 2 shows the STEG physical therapy intervention carried out for 3 weeks according to the recommendations of protocols delivered at some primary care centers in Spain. Participants with non-specific neck pain received a standardized Therapeutic Exercise Program as described by Häkkinen et al. [26]; participants with non-specific low back pain received a standardized Therapeutic Exercise Group Intervention as described by Albaladejo et al. [15]. At the end of each session, there was a group activity with the same Therapeutic Education Program described previously [24,25].

Each participant received a brochure with a detailed description of the standardized Therapeutic Exercise Program developed during the sessions with the same considerations as described before.

### 2.4. Statistical Procedure

Data analysis was performed for the description and comparison of sociodemographic and anthropometric characteristics of participants in both interventions (A_0_); percentage estimation of success achieved after each intervention (A_1_) and at week 12 (A_2_); evaluation of the effect achieved by each group after the intervention (A_1_) and at week 12 (A_2_) of HRQoL (SF-36v2) and non-specific neck and low back pain (VAS); and comparison between both groups at week 12 (A_2_).

Means, standard deviations, and frequencies were calculated. The chi-square test for qualitative variables, and Student’s *t*-test or Mann–Whitney U-test for quantitative variables; and the Friedman test for 3 parallel groups were used for comparisons of variables. Finally, four repeated measures multivariate models were carried out taking the scores obtained at A_0_, A_1_, and A_2_ for neck pain and low back pain (VAS); and PCS and MCS (SF-36v2) as dependent variables. Kolmogorov–Smirnov tests verified the normality of data. Significance level was accepted at 0.05. Data analysis was performed with SPSS^®^ version 26.

## 3. Results

### 3.1. Participant Flow

**Figure 1:**
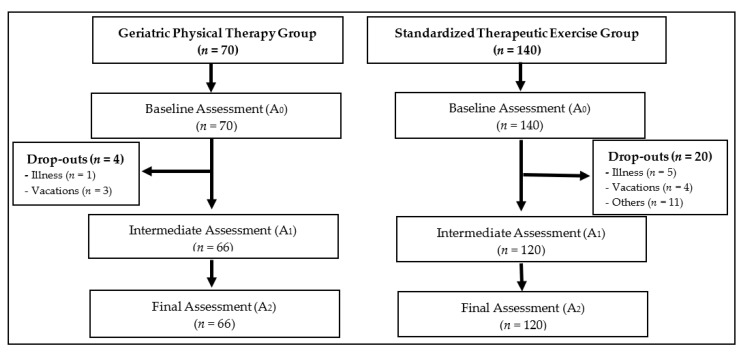
Participant flow according to the intervention groups.

A total of 70 subjects was included in the GPTG, and 66 of them (94%) finished the whole intervention. In the STEG, 140 subjects were included, and 120 (86%) of them finished the intervention planned for their group (Figure 1). All participants in both studies received the physical therapy program designed for each group intervention, and there were no changes from the previous studies plan. A post hoc power calculation was performed, and the final power of the study was 65%.

### 3.2. Baseline Data

Descriptive characteristics of the sample, according to the intervention groups, are shown in Table 3. There were no statistically significant differences (*p* > 0.05) between groups in all variables, except for employment status (*p* < 0.01).

There were statistically significant differences between groups in the SF-36v2 dimensions of PR (*p* < 0.01), GH (*p* = 0.01), VT (*p* = 0.03), and PCS (*p* = 0.02).

Concerning musculoskeletal pain measured with VAS, 38 participants (57.6%) referred non-specific neck pain, and 28 participants (42.4%) referred non-specific low back pain in the GPTG. In the STEG, 56 participants (46.7%) referred non-specific neck pain, and 64 participants (53.3%) referred non-specific low back pain. There was not a statistically significant difference between groups in non-specific low back pain (*p* = 0.28), although a statistically significant difference between groups was found in non-specific neck pain (*p* = 0.05).

### 3.3. Geriatric Physical Therapy Group Intervention

Table 4 shows the outcome values at baseline (A_0_), post-intervention (A_1_), and final (A_2_) assessments in the GPTG. There were statistically significant differences in BP (*p* = 0.02), non-specific neck pain (*p* < 0.01), and non-specific low back pain (*p* < 0.01) when comparing the assessments.

### 3.4. Standardized Therapeutic Exercise Group Intervention

Table 5 shows the outcome values at baseline (A_0_), post-intervention (A_1_), and final (A_2_) assessments in the STEG. There were statistically significant differences in all SF-36v2 dimensions (*p* < 0.01), except for ER (*p* = 0.34), non-specific neck pain (*p* < 0.01), and non-specific low back pain (*p* < 0.01) when comparing the assessments.

### 3.5. Comparison between Interventions: Repeated Measures Multivariate Models

PCS, MCS, and non-specific neck and low back pain values were considered in relation to the other variables (sex, age, marital status, work status, and body mass index) of the study for the multivariate analysis.

**Figure 2:**
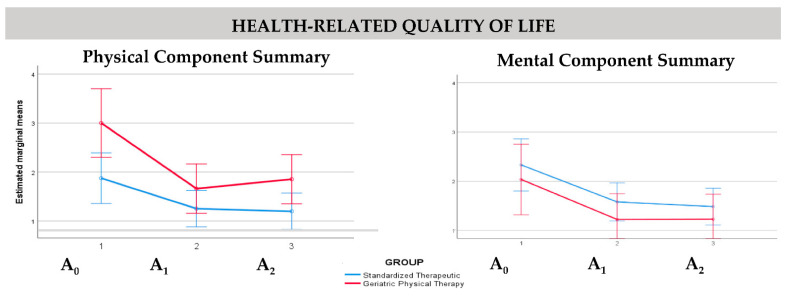
Estimated marginal means of health-related quality of life at baseline (A_0_), post-intervention (A_1_), and final (A_2_) assessments by intervention groups.

Regarding HRQoL, PCS values showed an association with age (*p* = 0.02) and BMI (*p* = 0.02) outcomes, while MCS values showed an association with sex (*p* = 0.04). Group intervention was not statistically significant—neither PCS nor MCS (*p* = 0.13 and *p* = 0.75, respectively). Figure 2 shows the estimated marginal means for HRQoL (PCS and MCS values) at baseline (A_0_), post-intervention (A_1_), and final (A_2_) assessments by intervention groups.

**Figure 3:**
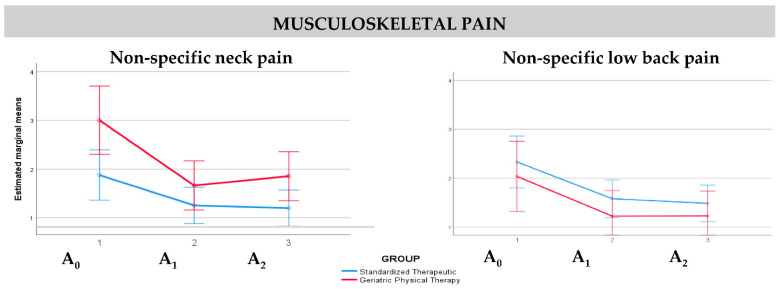
Estimated marginal means of musculoskeletal pain at baseline (A_0_), post-intervention (A_1_), and final (A_2_) assessments by intervention groups.

In relation to musculoskeletal pain, non-specific neck pain values showed an association with the intervention (*p* = 0.04), while non-specific low back pain values did not show an association with any other variables (*p* > 0.05). Figure 3 shows the estimated marginal means of musculoskeletal pain (non-specific neck pain and non-specific low back pain) at baseline (A_0_), post-intervention (A_1_), and final (A_2_) assessments by intervention groups.

### 3.6. Clinical Effectiveness in the Geriatric Physiotherapy Group and Standardized Therapeutic Exercise Group

Table 6 shows the clinical effectiveness of each intervention group at final assessment (A_2_) in relation to baseline assessment (A_0_). Comparing both interventions at week 12 (A_2_), there was a statistical significance only for non-specific neck pain (*p* = 0.02).

## 4. Discussion

Physical therapy interventions that include therapeutic exercise and therapeutic education programs as part of the interventions improve musculoskeletal pain in older adults, which may contribute to a better HRQoL perception and to a lower risk of disability and dependence in older adults [11,15,16,20,21,23,26,29,30,32]. Since no statistical significance was found between groups, one intervention could not prove to be more effective than the other. However, there was an MCID on non-specific neck pain intensity in favor of the GPTG. These results are similar to some studies comparing different physical therapy interventions for non-specific neck pain [16,26,27,28,29,30,31,32,33] and non-specific low back pain [15,34,35,36,37].

The GPTG intervention did not show significant improvement in any of the SF-36v2 dimensions, except for BP, although there was an MCID in the dimensions of SF, MH, and BP post-intervention (A_1_), and the latter was maintained at week 12 (A_2_). SF and MH are related to the degree to which physical and mental health problems interfere with the everyday social life of individuals. The intervention performed in the GPTG proved to be effective for reducing non-specific neck pain, and it seems to have had a positive influence on individuals′ social lives and physical and mental health perceptions.

Regarding the STEG intervention, all the outcomes showed significant improvement, except for ER, which refers to the degree to which emotional problems interfere with work or other activities of daily living. Baseline (A_1_) values for this dimension were high and probably less susceptible to changes. The findings in this group might be explained by the individuals′ employment situations in this group, who were still actively working.

Baseline HRQoL values were higher than Spanish reference values for people over 60 and very similar to general population values [39]. HRQoL perception is influenced by several aspects, such as, sex, age, genetic factors, body mass index, lifetime physical activity, and occupational position [23,44]. Silva et al. [45] performed a study with healthy older adults (≥60 years old) to investigate the relationship between health state perception and health predictors among the elderly from four European countries (Italy, Spain, Portugal, and Hungary). They concluded that age and country influence an individual’s QoL perception, and Spanish individuals presented the worst perception of HRQoL, which was related to worse performance of strength and agility tests and less physical activity practice. In this study, no specific physical activity data were collected, but most participants reported high values of the PF and PR dimensions. In both groups, the lowest SF-36 dimension reported was BP, which was much lower than reference values [39]. Among other reasons, BP is related to high BMI [6], and most participants were overweight or obese. In fact, BP was the dimension that reached MCID either at A_1_ or A_2_ in both groups, and it had the most positive variation along the study, especially in the GPTG.

Regarding musculoskeletal pain, at baseline (A_0_) in the GPTG there was a higher incidence of non-specific neck pain, the same findings as in the Spanish elderly population but opposite to elderly populations worldwide, where non-specific low back pain is the most frequent [6]. However, in the STEG there was a higher incidence of non-specific low back pain, so that this group resembles both the general population in Spain and the general and elderly population worldwide [6]. This fact might be explained by the employment situation in each group.

The study was developed according to different official institutional recommendation and evidence proved from different physical therapy interventions with the aim of improving QoL perception and reducing non-specific chronic neck and low back pain intensity in older adults [1,2,3,15,16,22,26,27,28,29,30,31,32,33,34,35,36,37].

At baseline, sociodemographic and anthropometric data were similar to most studies developed with older adults. In both groups, most participants were women, married, and retired [15,16,35,46,47,48].

Adherence and participation were high in both groups. In the literature, physical therapy interventions for musculoskeletal pain that achieve the best therapeutic adherence are those that are individually directed and supervised and include self-handling, education, and prevention aspects [47,48,49]. Jordan at al. [47] developed a systematic review to assess the effectiveness of different interventions to improve adherence to exercise therapy in people with chronic musculoskeletal pain and concluded that the type of exercise prescribed does not influence levels of adherence, and that the therapeutic exercise program should be based on patient preference and the current evidence regarding their effectiveness in relation to the patient’s complaint.

The limitations of the study are that groups were different at baseline, demonstrating non-homogeneity between them that might have conditioned the results. For this reason, repeated measures multivariate models were performed considering the lack of homogeneity from baseline. Moreover, the lack of a control group without any intervention could have added more information about the effectiveness of the interventions. However, the authors decided to treat all the participants because of the characteristics of the population.

The strengths of the present study include MCID in some primary outcomes, 12-week follow-up, two different treatments for non-specific neck and low back pain, and high adherence to and participation rates of treatment in both groups, which are elements of great interest for the population of the study.

## 5. Conclusions

Within the limitations of the study, it was possible to conclude that both physical therapy interventions showed a positive effect for reducing non-specific neck pain and low back pain in healthy older adults, which may contribute to health-related quality of life improvement.

The individualized intervention performed in the GPTG may be more recommendable for older people with non-specific neck pain, as there was a higher pain reduction than in the STEG.

## Figures and Tables

**Table 1:** Geriatric Physical Therapy Group intervention.

Week	Activities	Duration
1–5	Individualized Physical Therapy Intervention: - Non-specific neck pain: 20′ of massage therapy (gliding (effleurage), kneading (petrissage), friction-gliding in general neck and shoulders and neck lateral flexion, lateral rotation, and flexion passive stretch) [29]; and 40′ of an individualized therapeutic exercise program (progressive isometric neck strength exercises in flexion, extension, and rotation performed in sitting position; dynamic exercises for neck, shoulders, upper extremities and abdominal muscles) [26]; - Non-specific low back pain: 20′ of massage therapy (gliding (effleurage), kneading (petrissage), friction (circular only), vibration, rocking and jostling and holding of low back muscles) [34]; and 40′ of an individualized therapeutic exercise program (stretching and active exercises for the abdominal, lumbar and thoracic back extensors, psoas, ischiotibial, and pelvic muscles) [15].	60′
	Therapeutic Education Program group session: Conferences about being physically active and therapeutic exercise routines; pain neuroscience education; measures to improve health status and quality of life; postural hygiene; and preventive measures on risk of falls [24,25].	30′
	Home-based program: Individualized therapeutic exercise 4 days a week	30′
6–12	Home-based program: Individualized therapeutic exercise 4 days a week	30′

**Table 2:** Standardized Therapeutic Exercise Group intervention.

Week	Activities	Duration
1–3	Standardized Physical Therapy Intervention: - Non-specific neck pain: group standardized Therapeutic Exercise Program (progressive isometric neck strength exercises in flexion, extension, and rotation performed in sitting position; dynamic exercises for neck, shoulders, upper extremities, and abdominal muscles) [26]; - Non-specific low back pain: group standardized Therapeutic Exercise Program (stretching and active exercises for the abdominal, lumbar and thoracic back extensors, psoas, ischiotibial, and pelvic muscles) [15].	60′
	Therapeutic Education Program group session: Conferences about being physically active and Therapeutic Exercise routines; pain neuroscience education; measures to improve health status and quality of life; postural hygiene; and preventive measures on risk of falls [24,25].	30′
	Home based program: Standardized Therapeutic Exercise 4 days a week	30′
4–12	Home based program: Standardized Therapeutic Exercise 4 days a week	30′

**Table 3:** Descriptive characteristics of the sample according to the intervention groups at baseline (A_0_).

	Geriatric Physical Therapy Group (*n* = 70)	Standardized Therapeutic Exercise Group (*n* = 140)	*p*-Value
Age	65.87 ± 4.37	66.31 ± 5.66	0.54 *
Height (m)	1.62 ± 7.62	1.64 ± 7.67	0.07 *
Weight (kg)	73.56 ± 13.38	72.91 ± 12.05	0.72 *
Sex (%)			0.61 **
Women	62.9	66.4	
Men	37.1	33.6	
Marital Status (%)			0.91 **
Single	7.1	5	
Married	75.7	79.3	
Widow	14.3	12.9	
Divorced	2.9	2.9	
Work Status (%)			<0.01 **
Retired	62.9	46.4	
Active	12.9	32.9	
Housewife	24.3	20.7	
Body mass index (%)			0.76 **
Underweight	5.7	7.9	
Normal range	41.4	45.7	
Overweight	25.7	25	
Obesity	5.7	21	
Health-Related Quality of Life (SF-36v2)			
Physical Function	83.26 ± 15.79	78.81 ± 18.60	0.09 *
Physical Role	86.91 ± 19.30	81.17 ± 17.67	<0.01 ***
Bodily Pain	63.12 ± 24.53	60.76 ± 21.15	0.47 *
General Health	69.89 ± 18.77	63.19 ± 18.52	0.01 *
Vitality	71.70 ± 17.64	65.80 ± 18.42	0.03 *
Social Function	91.61 ± 16.71	88.15 ± 18.92	0.19 *
Emotional Role	90.95 ± 19.74	93.58 ± 13.79	0.26 *
Mental Health	78.93 ± 18.53	77.71 ± 17.07	0.64 *
PCS	48.69 ± 8.38	45.88 ± 7.54	0.02 ***
MCS	54.34 ± 9.39	54.31 ± 8.85	0.98 *
Musculoskeletal Pain (Visual Analogue Scale)			
Non-specific neck pain	5.57 ± 2.09	4.65 ± 2.16	0.05 *
Non-specific low back pain	5.74 ± 2.21	5.25 ± 1.84	0.28 *

PCS: Physical Component Summary; MCS: Mental Component Summary; * Student *t*-test; ** Chi-square test; *** Mann–Whitney U-test.

**Table 4:** Geriatric Physical Therapy Group at baseline (A_0_), post-intervention (A_1_), and final (A_2_) assessments.

Geriatric Physical Therapy Group (*n* = 70)	Baseline (A_0_)		Intermediate (A_1_)		Final (A_2_)		*p*-Value *
	Mean	SD	Mean	SD	Mean	SD	
Health-Related Quality of Life (SF-36v2)							
Physical Function	83.26	15.79	85.43	13.09	85.64	13.93	0.07
Physical Role	86.91	19.30	89.56	16.26	87.06	18.41	0.11
Bodily Pain	63.12	24.53	68.64	21.00	68.21	23.77	0.02
General Health	69.89	18.77	72.14	17.43	68.52	17.84	0.09
Vitality	71.70	17.64	73.85	17.88	72.42	20.43	0.19
Social Function	91.61	16.71	95.00	12.66	93.57	13.91	0.18
Emotional Role	90.95	19.74	93.93	15.14	91.55	18.08	0.39
Mental Health	78.93	18.53	82.64	15.31	81.07	17.60	0.34
PCS	48.69	8.38	49.65	7.19	49.39	8.11	0.37
MCS	54.34	9.39	56.32	7.34	54.94	9.74	0.71
Musculoskeletal Pain (Visual Analogue Scale)							
Non-specific neck pain	5.57	2.09	3.22	2.04	3.51	2.12	<0.01
Non-specific low back pain	5.74	2.21	3.74	2.24	3.74	2.22	<0.01

SD: standard deviation; PCS: Physical Component Summary; MCS: Mental Component Summary; * Friedman test.

**Table 5:** Standardized Therapeutic Exercise Group at baseline (A_0_), post-intervention (A_1_), and final (A_2_) assessments.

Standardized Therapeutic Exercise Group (*n* = 140)	Baseline (A_0_)		Intermediate (A_1_)		Final (A_2_)		*p*-Value *
	Mean	SD	Mean	SD	Mean	SD	
Health-Related Quality of Life (SF-36v2)							
Physical Function	78.81	18.60	80.75	16.71	80.70	16.94	<0.01
Physical Role	81.17	17.67	84.25	14.90	83.80	15.64	<0.01
Bodily Pain	60.76	21.15	66.58	19.51	67.80	21.37	<0.01
General Health	63.19	18.52	64.44	18.55	63.71	18.02	0.01
Vitality	65.80	18.42	69.37	16.53	69.33	17.18	<0.01
Social Function	88.15	18.92	90.11	17.38	89.31	17.89	0.01
Emotional Role	93.58	13.79	94.11	12.26	93.34	13.42	0.34
Mental Health	77.71	17.07	79.82	16.87	80.25	17.79	<0.01
PCS	45.88	7.54	47.32	6.76	47.34	6.91	<0.01
MCS	54.31	8.85	55.06	8.16	54.91	8.44	0.03
Musculoskeletal Pain (Visual Analogue Scale)							
Non-specific neck pain	4.65	2.16	3.24	2.18	3.15	2.09	<0.01
Non-specific low back pain	5.25	1.84	3.75	2.12	3.57	2.05	<0.01

SD: standard deviation; PCS: Physical Component Summary; MCS: Mental Component Summary; * Friedman test.

**Table 6:** Clinical effectiveness by intervention groups.

	GPTG (*n* = 66) %	STEG (*n* = 120) %	*p*-Value	OR	IC
Health-Related Quality of Life (SF-36v2)					
Physical Component Summary	24.6	32.5	0.26	0.68	0.34; 1.24
Mental Component Summary	27.7	20.8	0.29	1.45	0.72; 2.93
Musculoskeletal Pain (Visual Analogue Scale)					
Non-specific neck pain	34.8	19.2	0.02	2.25	1.14; 4.45
Non-specific low back pain	30.3	27.5	0.68	1.14	0.59; 2.22

GPTG: Geriatric Physical Therapy Group; STEG: Standardized Therapeutic Exercise Group; OR: odds ratio; CI: confidence interval.

## Data Availability

Data are available under justified request from the authors.
